# Bortezomib resistance mutations in PSMB5 determine response to second-generation proteasome inhibitors in multiple myeloma

**DOI:** 10.1038/s41375-020-0989-4

**Published:** 2020-07-20

**Authors:** Kira Allmeroth, Moritz Horn, Virginia Kroef, Stephan Miethe, Roman-Ulrich Müller, Martin S. Denzel

**Affiliations:** 1 Max Planck Institute for Biology of Ageing, Joseph-Stelzmann-Str. 9b, D-50931 Cologne, Germany; 2 Acus Laboratories GmbH c/o Max Planck Institute for Biology of Ageing, Joseph-Stelzmann-Str. 9b, D-50931 Cologne, Germany; 3 CECAD—Cluster of Excellence, University of Cologne, Joseph-Stelzmann-Str. 26, D-50931 Cologne, Germany; 4 Department II of Internal Medicine, Faculty of Medicine and University Hospital Cologne, University of Cologne, Kerpener Str. 62, D-50937 Cologne, Germany; 5 Center for Molecular Medicine Cologne (CMMC), University of Cologne, Robert-Koch-Str. 21, D-50931 Cologne, Germany

**Keywords:** Cancer genetics, Myeloma, Targeted therapies

## To the Editor:

Multiple myeloma (MM) is an incurable disease characterized by clonal expansion of malignant plasma cells in the bone marrow [1]. Although the advent of novel therapeutics, including proteasome inhibitors (PIs), has greatly enhanced patient outcome, relapse is common in MM even under maintenance therapy and the period of remission decreases with each iteration of therapy [2]. The PI bortezomib (PS-341, Velcade) is a first-line treatment for many patients [3]; however, re-exposure to bortezomib after relapse does usually not lead to a further response [4–6]. Bortezomib targets the proteasome subunit β type 5 (PSMB5) that harbors chymotrypsin-like proteolytic activity [7]. Recently, somatic *PSMB5* substitutions were identified in a bortezomib-treated MM patient [8], suggesting that resistance through *PSMB5* point mutations is clinically relevant. Like bortezomib, the second-line PIs ixazomib and carfilzomib [9, 10], as well as the investigational agent oprozomib [11], occupy the PSMB5 substrate binding pocket, interfering with the catalytic N-terminal threonine residue. Thus, in cases in which resistance to the first-line PI is due to *PSMB5* mutations there is significant risk for resistance to other PIs. Given the limited knowledge regarding shared and distinct resistance mechanisms between different PIs, there is no established protocol for an evidence-based sequential PI treatment of MM patients. Taken together, to close the gap in decision-making for individualized treatment, there is a need to identify all resistance-associated *PSMB5* point mutations, to understand their consequence for proteasomal activity, and to stratify the resistance patterns towards second-generation PIs.

To generate resistance-associated *PSMB5* point mutations for functional analysis, we used bortezomib selection in KMS-18 and KMS-27 MM cells that have a wild-type *PSMB5* locus. We identified spontaneous heterozygous mutations resulting in a PSMB5 T21A substitution in KMS-18 cells and a PSMB5 A49V substitution in KMS-27 cells (Supplementary Fig. 1a, b). The cells were bortezomib resistant (Supplementary Fig. 1c, d) and, surprisingly, showed no defects in proliferation or proteasome activity (Supplementary Fig. 1e–h). Apparently, the presence of the wild-type allele in cells with heterozygous *PSMB5* mutations precludes functional analysis.

**Fig. 1:**
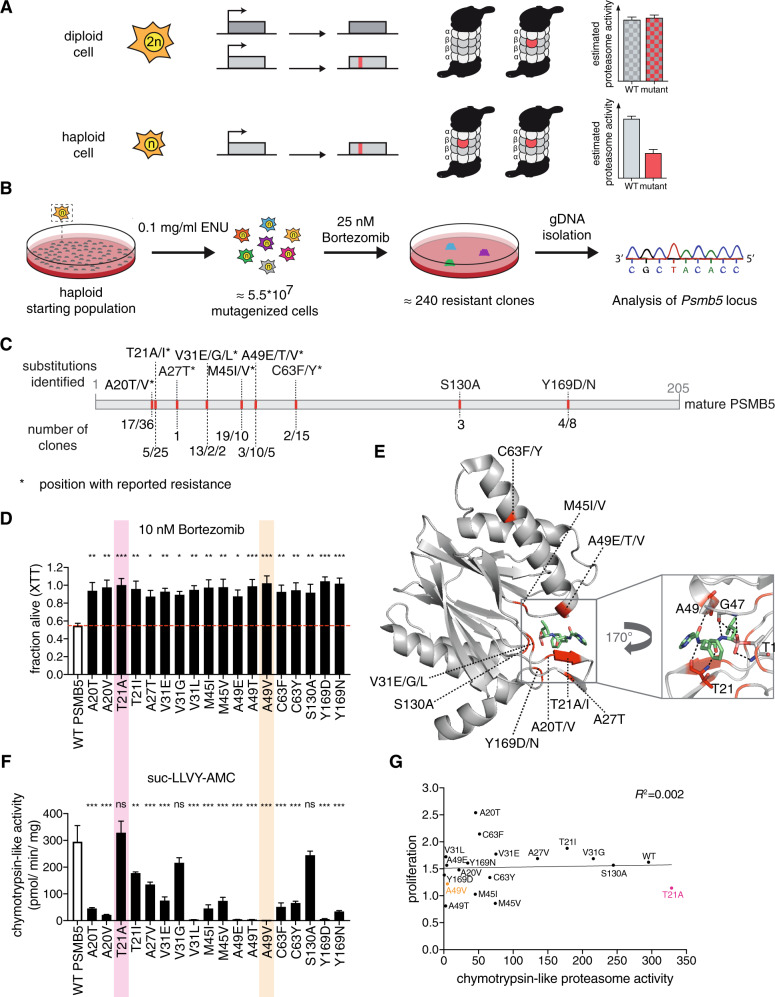
Unbiased identification and characterization of clinically relevant bortezomib resistance mutations in *Psmb5* using haploid cells. **a** Schematic representation of diploid cells, in which the presence of a wild-type allele masks the phenotype caused by a mutant allele. In haploid cells, there is a direct genotype–phenotype correlation, enabling functional analysis. **b** Schematic representation of experimental workflow for bortezomib resistance screen using ENU mutagenesis. **c** Schematic representation of PSMB5. Amino acid substitutions identified in the screen are highlighted in red. Positions with reported resistance are marked with an asterisk. For more information, see Supplementary Table 1. **d** Cell viability assay (XTT) of wild-type (WT) control cells and isolated clones treated with 10 nM bortezomib. Statistical significance was calculated by one-way ANOVA Dunnett’s post-hoc test. ***p < 0.001, **p < 0.01, *p < 0.05, ns not significant. Mean + SEM (*n* = 4). **e** Crystal structure of human PSMB5 (gray) in complex with bortezomib (green). Identified substitutions are highlighted in red. Hydrogen bonds between bortezomib and the amino acids in the binding pocket are shown (black dashed lines). PDB: 5LF3. **f** Chymotrypsin-like proteasome activity of wild-type and CRISPR/Cas9-engineered AN3-12 cells with the indicated PSMB5 substitutions using suc-LLVY-AMC as a substrate. Mean + SEM (*n* = 3). **g** Correlation of mean chymotrypsin**-**like activity (Fig. 1f) with mean proliferation on day 3 (Supplementary Fig. 2e) of wild-type and CRISPR/Cas9-engineered AN3-12 cells with the indicated PSMB5 substitutions. *R*^2^ was calculated by linear regression fit using GraphPad Prism.

In contrast to di- or polyploid cells, both recessive and dominant mutations lead to a phenotype in haploid cells, and it is possible to analyze the functional consequences of a mutation without the interference of the remaining wild-type allele (Fig. 1a). In an unbiased forward genetic approach using N-ethyl-N-nitrosourea mutagenesis, we screened 55 million haploid cells for resistance to 25 nM bortezomib. The *Psmb5* locus of 201 randomly selected resistant colonies was sequenced (Fig. 1b) and we identified *Psmb5* mutations in 181 lines, resulting in 18 distinct amino acid substitutions at 9 positions (Fig. 1c and Supplementary Table 1). From the isolated colonies, we generated individual mutant cell lines that were up to twofold more resistant to 10 nM bortezomib compared to the wild-type control (Fig. 1d), while proliferation was largely unaffected (Supplementary Fig. 2a). In addition to known resistance mutations [8, 12, 13], we found two residues that had not been implicated in bortezomib resistance before (S130, Y169). All identified amino acid substitutions, with the exception of C63F/Y, cluster in the bortezomib binding pocket of PSMB5 (Fig. 1e). Taken together, our mutagenesis approach confirmed known mutations, and also identified novel bortezomib resistance alleles in *Psmb5* at high saturation. It has yielded the largest set of *PSMB5* resistance mutations reported so far.

We introduced the individual *Psmb5* mutations in wild-type AN3-12 haploid cells and found that they again led to bortezomib resistance (Supplementary Table 2 and Supplementary Fig. 2b). Thus, the mutagenesis approach is a powerful tool to faithfully mimic clonal evolution in vitro. To elucidate the effect of the substitutions on proteolysis, we next performed proteasome activity assays. Consistent with active site mutations, the chymotrypsin-like activity of the β5 subunit was dramatically decreased in many of the homozygous mutant cells (Fig. 1f), except for the mutant cells with PSMB5 T21A, V31G, and S130A substitutions. Caspase-like and trypsin-like proteasome activities were not changed in any of the engineered *Psmb5* mutant cell lines (Supplementary Fig. 2c, d). Surprisingly, although chymotrypsin-like activity was severely reduced by many substitutions, proliferation was largely unaffected in most mutant cell lines (Supplementary Fig. 2e). Despite very low residual chymotrypsin-like catalytic activity, the A20T substitution even enhanced proliferation. Only the M45 and A49T substitutions reduced cell growth. Importantly, the use of haploid cells revealed that diminished proteasome activity did not correlate with poor proliferation under normal growth conditions (*R*^2^ = 0.002; Fig. 1g). MM patient cells are heterozygous for a given mutation and the wild-type allele is sufficient to rescue proteasome activity, as shown in cultured MM cells (Supplementary Fig. 1g, h). While the A49V substitution caused a complete loss of chymotrypsin-like activity in the stem cells, MM cells heterozygous for the mutation resulting in PSMB5 A49V showed unchanged proteasome activity. A mutant *PSMB5* allele can thus endow PI resistance during treatment, while the remaining unaltered copy of the gene provides full fitness upon PI removal. Overall, our data indicate that mutant plasma cell clones are likely to persist without selective PI pressure. It is, therefore, unlikely that the rare detection of *PSMB5* variants in patients after PI treatment is due to reduced proliferation.

**Fig. 2:**
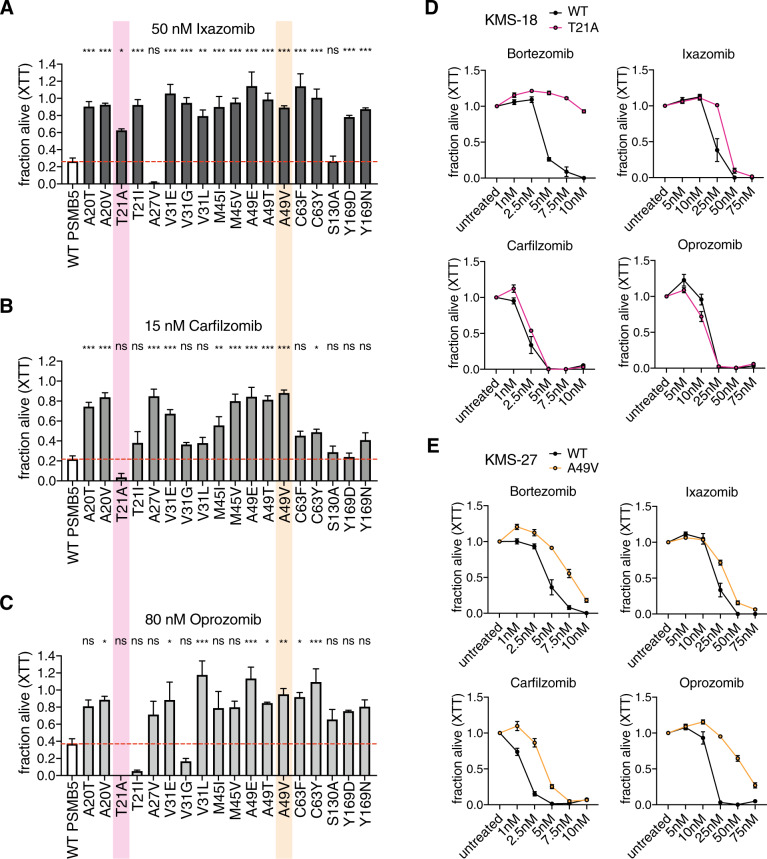
Bortezomib-resistant *Psmb5* mutant cells display differential response to second-generation proteasome inhibitors. **a** Cell viability of wild-type and CRISPR/Cas9-engineered AN3-12 cells with the indicated PSMB5 substitutions treated with 50 nM ixazomib. Mean + SEM (*n* = 5; *n* = 3 for V31L). **b** Cell viability of wild-type and CRISPR/Cas9-engineered AN3-12 cells with the indicated PSMB5 substitutions treated with 15 nM carfilzomib. Mean + SEM (*n* = 5; *n* = 3 for V31L). **c** Cell viability of wild-type CRISPR/Cas9-engineered AN3-12 cells with the indicated PSMB5 substitutions treated with 80 nM oprozomib. Mean + SEM (*n* = 3). **a**–**c** T21A is highlighted in pink and A49V is highlighted in orange. Statistical significance was calculated by one-way ANOVA Dunnett’s post-hoc test. ***p < 0.001, **p < 0.01, *p < 0.05, ns not significant. **d** Cell viability of wild-type (black) and PSMB5 T21A KMS-18 cells (pink) treated with the indicated PIs. **e** Cell viability of wild-type (black) and PSMB5 A49V KMS-27 cells (orange) treated with the indicated PIs. **d**, **e** Data are presented as mean ± S**E**M (*n* = 3).

To elucidate the consequence of acquired bortezomib resistance regarding the effectiveness of second-line PIs, we treated the engineered AN3-12 cells carrying individual PSMB5 substitutions with ixazomib, carfilzomib, or oprozomib. Bortezomib and ixazomib are boronic acids, while carfilzomib and oprozomib belong to the bulkier epoxyketones (Supplementary Fig. 3a). With the exception of A27V and S130A substitutions, bortezomib-resistant PSMB5 mutant cell lines were also resistant to 50 nM ixazomib (Fig. 2a), consistent with the compounds’ structural similarities. In contrast, treatment with 15 nM carfilzomib or 80 nM oprozomib resulted in varying degrees of resistance (Fig. 2b, c). While T21 substitutions displayed carfilzomib and oprozomib hypersensitivity, A49 mutation caused resistance to all PIs tested in this study. Notably, the *Psmb5* mutant clones isolated from the initial screen showed the same resistance patterns (Supplementary Fig. 3b–d).

Modeling of the T21A and A49V substitutions in the PSMB5 structure in complex with the different PIs [14, 15] revealed changes in the binding pocket that help to explain the varying PI effectiveness: since alanine has a smaller side chain than threonine, the T21A substitution enlarges the binding pocket, potentially reducing bortezomib and ixazomib affinities (Supplementary Fig. 4a). However, the T21A mutants were hypersensitive to the irreversibly binding PIs carfilzomib and oprozomib, potentially due to better accessibility of the catalytic N-terminal threonine residue for the bulkier epoxyketones. In addition, we observed unchanged chymotrypsin-like activity in T21A mutants (Fig. 1f), supporting the notion that the binding pocket remained accessible to peptide substrates. In contrast to T21A, the replacement of alanine 49 with the larger valine caused steric clashes with all PIs and additionally with S129 of the β6 subunit of the proteasome (indicated by red disks in Supplementary Fig. 4b). This steric hindrance likely prevented PI binding to the PSMB5 active site. Also, the A49V substitution completely blunted the chymotrypsin-like activity (Fig. 1f), suggesting that substrates were unable to access the altered binding pocket. Thus, we identified two distinct mechanisms of PI resistance, which are supported by the structure of PSMB5. We went on to validate our findings from the haploid system in patient-derived MM cell lines: while the T21A substitution led to selective bortezomib and ixazomib resistance (Fig. 2d), A49V mutant MM cells were resistant to all PIs tested (Fig. 2e). As in mouse haploid cells, T21A mutants remained sensitive to carfilzomib and oprozomib (Fig. 2d). These data confirm our observations in the haploid cells regarding the differential response of the substitutions to second-generation PIs, highlighting their potential clinical relevance.

Finally, we clustered the mutants according to their level of resistance (Supplementary Fig. 5): A20, M45, and A49 substitutions were resistant to all tested PIs, with slight variabilities regarding carfilzomib. Mutations in C63 or Y169 resulted in a partially maintained response to carfilzomib, implicating carfilzomib as a potential treatment option for patients with these substitutions. Furthermore, the resistance pattern of T21 substitutions suggests carfilzomib and oprozomib as the possible second-line agents of choice. In general, the affected amino acid position in PSMB5 appeared more important than the type of substitution. Overall, among the tested compounds, carfilzomib was the most efficient PI to overcome acquired bortezomib resistance.

Taken together, our results begin to explain why acquired bortezomib resistance might influence second-generation PI treatments in MM patients. However, the true frequency of *PSMB5* mutations in malignant plasma cell clones is still unclear. Due to the heterogeneity of the disease, so far only one study identified *PSMB5* mutations in a patient [8]. Based on our results, the rare detection of *PSMB5* variants in patients after PI treatment is not due to reduced proliferation of mutant plasma cell clones; instead, the remaining wild-type copy of the *PSMB5* gene provides full fitness upon PI removal. Therefore, further in-depth analysis of the *PSMB5* status, especially in relapsed MM patients, is of utmost priority. In the future, repetitive bone marrow sampling might guide the treatment of MM disease and our study suggests that this approach ought to be included in future clinical trials. Over the long-term, we hope that patient stratification and subsequent treatment with the efficacious drug of choice will become the state-of-the-art in MM.

## Supplementary information

Supplementary material
