# Supplementary material for: Bortezomib resistance mutations in PSMB5 determine response to second-generation proteasome inhibitors in multiple myeloma
Source: Leukemia. 2020 Jul 20;35(3):887–92. doi: 10.1038/s41375-020-0989-4 (PMC7932915; doi:10.1038/s41375-020-0989-4)
Supplement: Supplementary file 1 — Supplementary material [file 41375_2020_989_MOESM1_ESM.docx]

**Supplementary information**

**Bortezomib resistance mutations in PSMB5 determine response to second-generation proteasome inhibitors in multiple myeloma**

Short Title: PSMB5 variants in proteasome inhibitor resistance

Kira Allmeroth^1^, Moritz Horn^2^, Virginia Kroef^1^, Stephan Miethe^1^, Roman-Ulrich Müller^3,4,5^ and Martin S. Denzel^1,3,5^*

^1^Max Planck Institute for Biology of Ageing

Joseph-Stelzmann-Str. 9b

D-50931 Cologne, Germany

^2^Acus Laboratories GmbH

c/o Max Planck Institute for Biology of Ageing

Joseph-Stelzmann-Str. 9b

D-50931 Cologne, Germany

^3^CECAD - Cluster of Excellence

University of Cologne

Joseph-Stelzmann-Str. 26

D-50931 Cologne, Germany

^4^Department II of Internal Medicine

Faculty of Medicine and University Hospital Cologne

University of Cologne

Kerpener Str. 62

D-50937 Cologne, Germany

^5^Center for Molecular Medicine Cologne (CMMC)

University of Cologne

Robert-Koch-Str. 21

D-50931 Cologne, Germany

*Corresponding author: Martin S. Denzel

E-Mail: martin.denzel@age.mpg.de

phone: +49-(0)221 379 70 443

fax: +49-(0)221 379 70 88-443**Supplementary Methods**

**Cell lines and culture conditions**

AN3-12 mouse haploid embryonic stem cells were cultured as previously described (1). In brief, cells were grown in DMEM high glucose (Sigma-Aldrich, St. Louis, Missouri) supplemented with 15% fetal bovine serum, penicillin/streptomycin, glutamine, non-essential amino acids, sodium pyruvate (all Thermo Fisher Scientific, Waltham, Massachusetts), β-mercaptoethanol, and LIF (both Merck Millipore, Darmstadt, Germany) on non-coated tissue culture plates.

The human cell line KMS-18 was established from a 58-year-old male with MM associated with hyperammonemia (2). The authors identified chromosomal abnormalities, including add(1)(q32), add(10)(q24) and add(17)(p11) and the translocation t(4;14)(p16.3;q32.3). The cell line KMS-27 was derived from peripheral blood of a 52-year-old male MM patient. This cell line was first described in 2003 (3) and has the translocation t(11;14)(q16.3;q32). The MM cell lines were cultured in RPMI-1640 (Thermo Fisher Scientific), supplemented with 15% fetal bovine serum, glutamine (both Thermo Fisher Scientific), and gentamicin (Sigma-Aldrich). KMS-18 and KMS-27 cells were selected with 10 nM or 7.5 nM bortezomib, respectively. Resistant colonies emerged from spontaneously mutated single cells after approximately four weeks of selection, were transferred to 24-well plates and the *PSMB5* locus was analyzed. A separate cell line was established from each colony. DNA was extracted (QuickExtract DNA extraction solution, Lucigen, Middleton, Wisconsin) and exon 2 of the *PSMB5* gene was specifically amplified by PCR using the primers huexon2_fwd (ACGGAGAAAGAGATGTGCTGG), and huexon2_rev (AGCATTGACACCAAGCCCTT). Sanger sequencing was performed at Eurofins Genomics GmbH (Ebersberg, Germany).

**Cell sorting**

AN3-12 cells were stained with 10 µg/ml Hoechst 33342 (Thermo Fisher Scientific) for 30 min at 37 °C. Propidium iodide (Sigma-Aldrich) was used to exclude dead cells. Haploid or diploid cells were sorted using a FACSAria Fusion sorter and flow profiles were recorded with the FACSDiva software (BD, Franklin Lakes, New Jersey).

**Chemical mutagenesis**

Chemical mutagenesis was performed as previously described (4). In brief, AN3-12 cells were incubated with 0.1 mg/ml ENU for two hours at room temperature, washed five times with LIF-free medium, and plated on culture dishes. The cells were selected with 25 nM Bortezomib (Selleckchem, Houston, Texas) for three weeks, starting 24 h after mutagenesis. Resistant colonies emerging from mutagenized single cells were transferred to 24-well plates and the *Psmb5* locus was analyzed. A separate cell line was established from each colony. DNA was extracted (QuickExtract DNA extraction solution, Lucigen) and exon 2 or exon 3 of the *Psmb5* gene were specifically amplified by PCR using the primers exon2_fwd (GTATTTGTGGTCTTACGGGGC), exon2_rev (AACCAGTTCCCAGATGAAGAAA), exon3_fwd (GGGTGGTGTGTGTGAGAGAG), and exon3_rev (CCAGGGTTCGGGGGAGATAT) before sequencing.

**Cell viability assay**

Cell viability was analyzed using the XTT cell proliferation Kit II (Roche Diagnostics, Basel, Switzerland). Drug treatment was started 24 h after cell seeding. Absorbance was measured after 72 h of treatment and normalized to the respective untreated controls (technical duplicates). The absorbance of untreated wells was plotted to investigate proliferation. The mean of independent biological replicates is displayed. Mitomycin C (4 µg/ml) was used to inhibit proliferation.

**CRISPR/Cas9-mediated gene editing**

*Psmb5* mutations were engineered in haploid wildtype AN3-12 cells using the CRISPR/Cas9 system (5). Sequences of small guide RNAs were designed online (http://crispor.org, Supplementary Table 2) and were purchased from Sigma-Aldrich. The guide RNAs matching the following criteria were cloned into the Cas9-GFP expressing plasmid PX458 (Addgene #48138): high score, minimal number of off-targets, maximum of 60 bp distance from the PAM site to the position of interest. To generate the different *Psmb5* mutant cell lines, respective combinations of guide RNA-Cas9-GFP expressing plasmids and the corresponding single stranded DNA repair template (Sigma-Aldrich, Supplementary Table 2) were transfected using Lipofectamine 2000 (Thermo Fisher Scientific). The position of interest was located approximately in the middle of the repair template comprising 120 bp. The PAM sites of the guide RNAs were mutated in the repair template, if possible. Cells were transferred to 10 cm plates 24 h post transfection and selected with 25 nM bortezomib for 2 weeks. This selection enriches for successfully engineered cells. Resistant colonies emerging from single cells were picked and analyzed. DNA was extracted (QuickExtract DNA extraction solution, Lucigen) and exon 2 or exon 3 of the *Psmb5* gene were amplified by PCR using the primers described above. Positive clones detected by Sanger sequencing were sorted diploid prior to further experiments. We successfully generated all individual PSMB5 substitutions except for A27T. The A27T mutant line from the screen displayed growth retardation, which probably interfered with our approach. Instead, we used mutant cells with an A27V substitution. The A27V and A49V *Psmb5* mutant cell lines were generated in a previous study (4).

**Proteasome activity assay**

The three proteolytic activities of the proteasome were assessed by measuring the rate of hydrolysis of fluorogenic peptides: chymotrypsin-like (suc-LLVY-AMC, Sigma-Aldrich), trypsin-like (ac-RLR-AMC, Enzo Life Sciences, Farmingdale, New York), and caspase-like activity (Z-LLE-AMC, Enzo Life Sciences). Cell extracts were prepared in 25 mM Tris HCl pH 7.5 by sonication. The protein concentration was determined using the Pierce^TM^ BCA protein assay kit (ThermoFisher Scientific). 20 µg lysate (technical triplicates) were incubated with 12.5 µM of the respective fluorogenic peptide in a total volume of 200 µl. AMC fluorescence was measured using 355 nm excitation and 460 nm emission filters with free AMC (Sigma-Aldrich) as standard every min for 30 min at 37 °C. The mean of independent biological replicates is displayed.

**Structural analysis**

Crystal structures of human PSMB5 in complex with the different PIs used in this study were previously published (6, 7). PDB accession codes: 5LF3 (bortezomib), 5LF7 (ixazomib), 4R67 (carfilzomib), 5LEZ (oprozomib). Structure figures were generated using PyMOL (The PyMOL Molecular Graphics System, Version 2.1.1, Schrödinger, LLC). The mutagenesis tool of PyMOL was used to model the substitutions. Van-der-Waals clashes (steric hindrance, red discs) were visualized using the PyMOL plugin show_bumps.

**Statistical analysis**

Data are presented as mean ± SEM or as mean + SEM. The mean of technical replicates is plotted for each biological replicate. Biological replicates represent different passages of the cells that were seeded on independent days. Statistical significance was calculated using GraphPad Prism (GraphPad Software, San Diego, California). The statistical test used is indicated in the respective figure legend. Significance levels are * p<0.05, ** p<0.01, *** p<0.001 versus the respective control.

**Supplementary Table 1** shows the *PSMB5* mutations identified in the bortezomib resistance screen and the corresponding amino acid substitutions.

**Supplementary Table 2** contains the guide RNAs and repair template sequences.

**Supplementary Figure 1** shows the characterization of *PSMB5* mutant MM cell lines.

**Supplementary Figure 2** provides information about the functional characterization of *Psmb5* mutant AN3-12 cells.

**Supplementary Figure 3** displays XTT viability assays using the cell clones identified in the resistance screen.

**Supplementary Figure 4** provides structural information about the T21A and A49V substitutions of PSMB5.

**Supplementary Figure 5** shows the values of the XTT viability assay (Figure 2) as a heat map.

**Supplementary Table 1: Overview of the identified mutations in PSMB5 and their consequence at the amino acid level.**

| Position | Wildtype nucleotide | Mutant nucleotide | Amino acid change | Mature PSMB5 | % of clones | Guides  used |
| --- | --- | --- | --- | --- | --- | --- |
| 235 | C | T | A79T | A20T | 9.4 | 1;2;3 |
| 236 | G | A | A79V | A20V | 19.9 | 1;2;3 |
| 238 | T | C | T80A | T21A | 2.8 | 1;2;3 |
| 239 | G | A | T80I | T21I | 13.8 | 1;2;3 |
| 256 | C | T | A86T | A27T | 0.6 | 1;2;3 |
| 268 | C | A | V90L | V31L | 1.1 | 1;2;3 |
| 269 | A | T | V90E | V31E | 7.2 | 1;2;3 |
| 269 | A | C | V90G | V31G | 1.1 | 1;2;3 |
| 310 | T | C | M104V | M45V | 10.5 | 2;3;4 |
| 312 | C | A | M104I | M45I | 5.5 | 2;3;4 |
| 322 | C | T | A108T | A49T | 5.5 | 2;3;4 |
| 323 | G | T | A108E | A49E | 1.7 | 2;3;4 |
| 323 | G | A | A108V | A49V | 2.8 | 2;3;4 |
| 365 | C | A | C122F | C63F | 1.1 | 4;5 |
| 365 | C | T | C122Y | C63Y | 8.3 | 4;5 |
| 468 | A | C | S189A | S130A | 1.7 | 6;7 |
| 565 | A | C | Y228D | Y169D | 2.2 | 8;9 |
| 682 | A | T | Y228N | Y169N | 4.4 | 8;9 |

**Supplementary Table 2: Sequences of the guide RNAs and repair templates used for CRISPR/Cas9-mediated engineering.**

|  | sequence |
| --- | --- |
| Guide 1 | GACCTTAACTAGTTTCTCCA |
| Guide 2 | GAGAGATCAACCCGTACCTTC |
| Guide 3 | GCCATGGTGCCCAGAAGGTA |
| Guide 4 | GCAGCTTCTGGGAGCGGTTGT |
| Guide 5 | CATGTTAGCGAGCAGTTTGG |
| Guide 6 | GTCTACGTAGTAGAGGCCTG |
| Guide 7 | CGTGTATGCTTACGGCGTTA |
| Guide 8 | GGCTCGGCGGGCCAGATCAT |
| Guide 9 | TCCAGCCATCCTCCCGCACG |
| Repair template A20T | CATCGTGCCGAGGAGGTACGGGTTTATCTCTATTACTTTCTTCACCGTCTGGGAAGCAATATAAGCACCTGCTGTGGTCCGGGAATCCGCTGCAACAATGACTCCATGCAGGAACTA |
| Repair template A20V | CATCGTGCCGAGGAGGTACGGGTTTATCTCTATTACTTTCTTCACCGTCTGGGAAGCAATATAAGCACCTGCTGTGACCCGGGAATCCGCTGCAACAATGACTCCATGCAGGAACTA |
| Repair template T21A | CATCGTGCCGAGGAGGTACGGGTTTATCTCTATTACTTTCTTCACCGTCTGGGAAGCAATATAAGCACCTGCTGCGGCCCGGGAATCCGCTGCAACAATGACTCCATGCAGGAACTA |
| Repair template T21I | GCTGCAATCCGCTGCACCCCCAGCCATCGTGCCGAGGAGGTACGGGTTTATCTCTATTACTTTCTTCACCGTCTGGGAAGCAATATAAGCACCTGCTATGGCCCGGGAATCCGCTGCAACAATGACTCCATGCAGGAACTAGTTGAGGTCAGAGAAAACAAAAAGAGAGGCCCCGTAAGACCAC |
| Repair template V31E | CATCGTGCCGAGGAGGTACGGGTTTATCTCTATTACTTTCTTCTCCGTCTGGGAAGCAATATAAGCACCTGCTGTGGCCCGGGAATCCGCTGCAACAATGACTCCATGCAGGAACTA |
| Repair template V31G | CATCGTGCCGAGGAGGTACGGGTTTATCTCTATTACTTTCTTCCCCGTCTGGGAAGCAATATAAGCACCTGCTGTGGCCCGGGAATCCGCTGCAACAATGACTCCATGCAGGAACTA |
| Repair template V31L | CATCGTGCCGAGGAGGTACGGGTTTATCTCTATTACTTTCTTCAACGTCTGGGAAGCAATATAAGCACCTGCTGTGGCCCGGGAATCCGCTGCAACAATGACTCCATGCAGGAACTA |
| Repair template M45I | AGCTCATAGATTCGACACTGCCGAGCTAACAAGCGCTCCCAGAAACTGCAATCCGCTGCACCCCCAGCAATGGTGCCGAGGAGGTACGGGTTTATCTCTATTACTTTCTTCACCGTCTGG |
| Repair template M45V | AGCTCATAGATTCGACACTGCCGAGCTAACAAGCGCTCCCAGAAACTGCAATCCGCTGCACCCCCAGCCACGGTGCCGAGGAGGTACGGGTTTATCTCTATTACTTTCTTCACCGTCTGG |
| Repair template A49E | AGCTCATAGATTCGACACTGCCGAGCTAACAAGCGCTCCCAGAAACTGCAATCCGCTTCACCCCCAGCCATCGTGCCGAGGAGGTACGGGTTTATCTCTATTACTTTCTTCACCGTCTGG |
| Repair template A49T | AGCTCATAGATTCGACACTGCCGAGCTAACAAGCGCTCCCAGAAACTGCAATCCGCTGTACCCCCAGCCATCGTGCCGAGGAGGTACGGGTTTATCTCTATTACTTTCTTCACCGTCTGG |
| Repair template C63F | TGATACACCATATTAGCGAGCAGCTTGGATGCTGCTGCGACCGAGATGCGTTCCTTATTGCGAAGCTCATAGATTCGAAACTGCCGAGCTAACAATCGCTCCCAGAAACTGCAATCCGCT |
| Repair template C63Y | TGATACACCATATTAGCGAGCAGCTTGGATGCTGCTGCGACCGAGATGCGTTCCTTATTGCGAAGCTCATAGATTCGATACTGCCGAGCTAACAATCGCTCCCAGAAACTGCAATCCGCT |
| Repair template S130A | AGTAGCCTCGATCCATTACGCCATAAGCATAGACGGAGCCAGCGCCCACTGAGAAGGCGGTCCCAGAGATCCTGTTCCCCTCGCTATCTACATAGTACAGGCCTGAGGGGATGAGGTGAG |
| Repair template Y169D | TGGACACCCGGATCCATCCATCCTCCCGGACGTGATAGAGGTTGACTGCCCCTCCGGAGTCGGCATCTCTGTAGGTGGCTTGGTAGATGGCGCGGCGGGCCAGGTCATAAGCCTCCTCCA |
| Repair template Y169N | TGGACACCCGGATCCATCCATCCTCCCGGACGTGATAGAGGTTGACTGCCCCTCCGGAGTTGGCATCTCTGTAGGTGGCTTGGTAGATGGCGCGGCGGGCCAGGTCATAAGCCTCCTCCA |


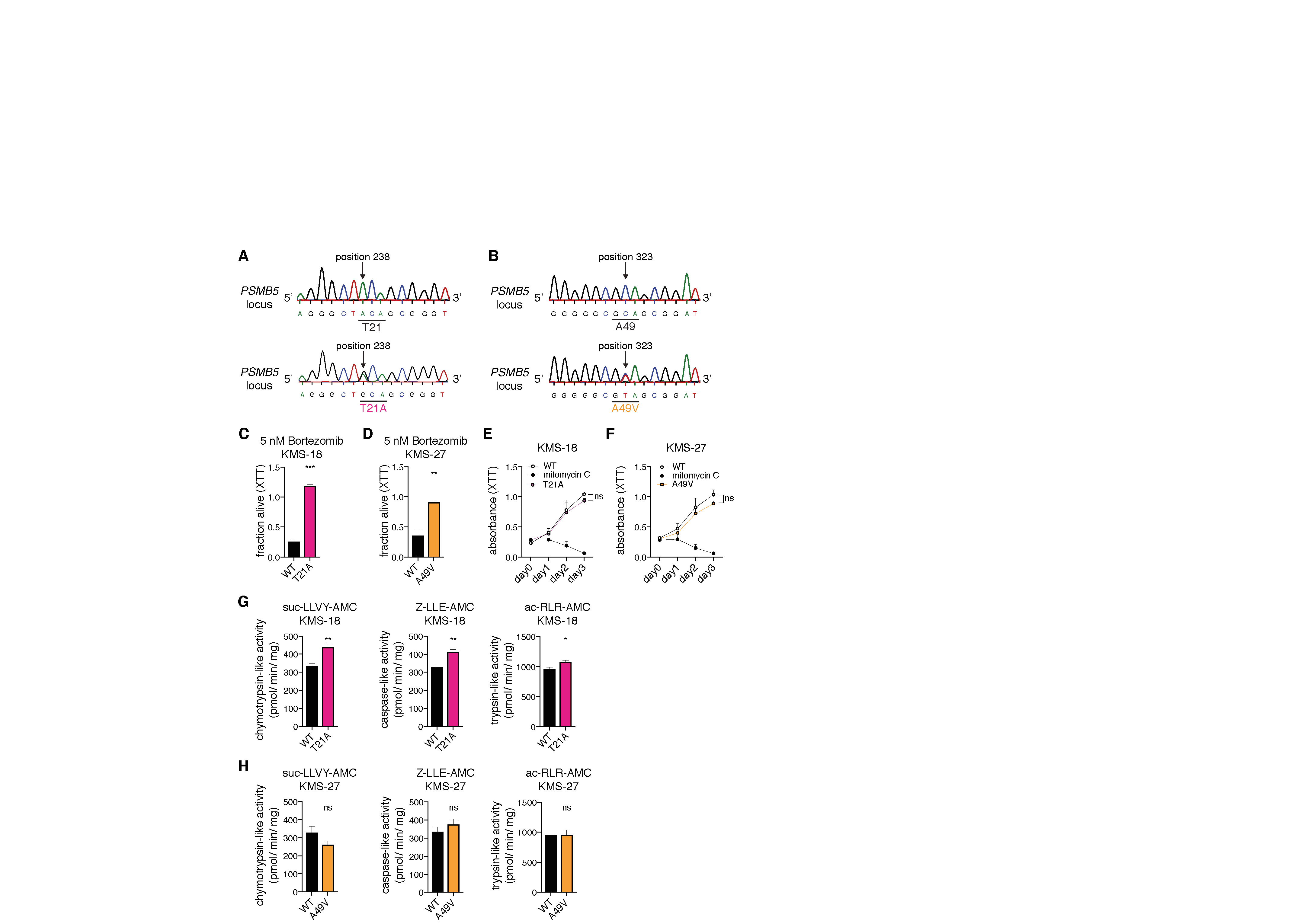


**Supplementary Figure 1: Heterozygous PSMB5 mutations in MM cells elude functional analysis.** (**A**) Sanger sequencing of PSMB5 locus in wildtype (WT) KMS-18 cells (top) and from KMS-18 cells with the heterozygous A to G transition at position 238 resulting in the T21A substitution after bortezomib selection (bottom). For more information, see Supplementary Table 1. (**B**) Sanger sequencing of PSMB5 locus in wildtype KMS-27 cells (top) and from KMS-27 cells with the heterozygous C to T transition at position 323 resulting in the A49V substitution after bortezomib selection (bottom). For more information, see Supplementary Table 1. (**C**) Cell viability of KMS-18 wildtype and mutant cells treated with 5 nM bortezomib. (**D**) Cell viability of KMS-27 wildtype and mutant cells treated with 5 nM bortezomib. (**E**) Proliferation of wildtype KMS-18 cells and mutant KMS-18 cells with the PSMB5 T21A substitution assessed by cell viability assay. Mitomycin C treatment served as control. (**F**) Proliferation of wildtype KMS-27 cells and mutant KMS-27 cells with the PSMB5 A49V substitution assessed by cell viability assay. Mitomycin C treatment served as control. (**G**) Chymotrypsin-like, caspase-like, and trypsin-like proteasome activity of KMS-18 wildtype and mutant cells. (**H**) Chymotrypsin-like, caspase-like, and trypsin-like proteasome activity of KMS-27 wildtype and mutant cells. (**C**-**D**, **G**-**H**) Statistical significance was calculated by unpaired t-test. Two-tailed p-values: *** p<0.001, ** p<0.01, * p<0.05, ns: not significant. Mean + SEM (n=3). (**E**-**F**) Statistical significance was calculated by Two-way ANOVA, Fisher’s LSD test, ns: not significant. Mean + SEM (n=2).


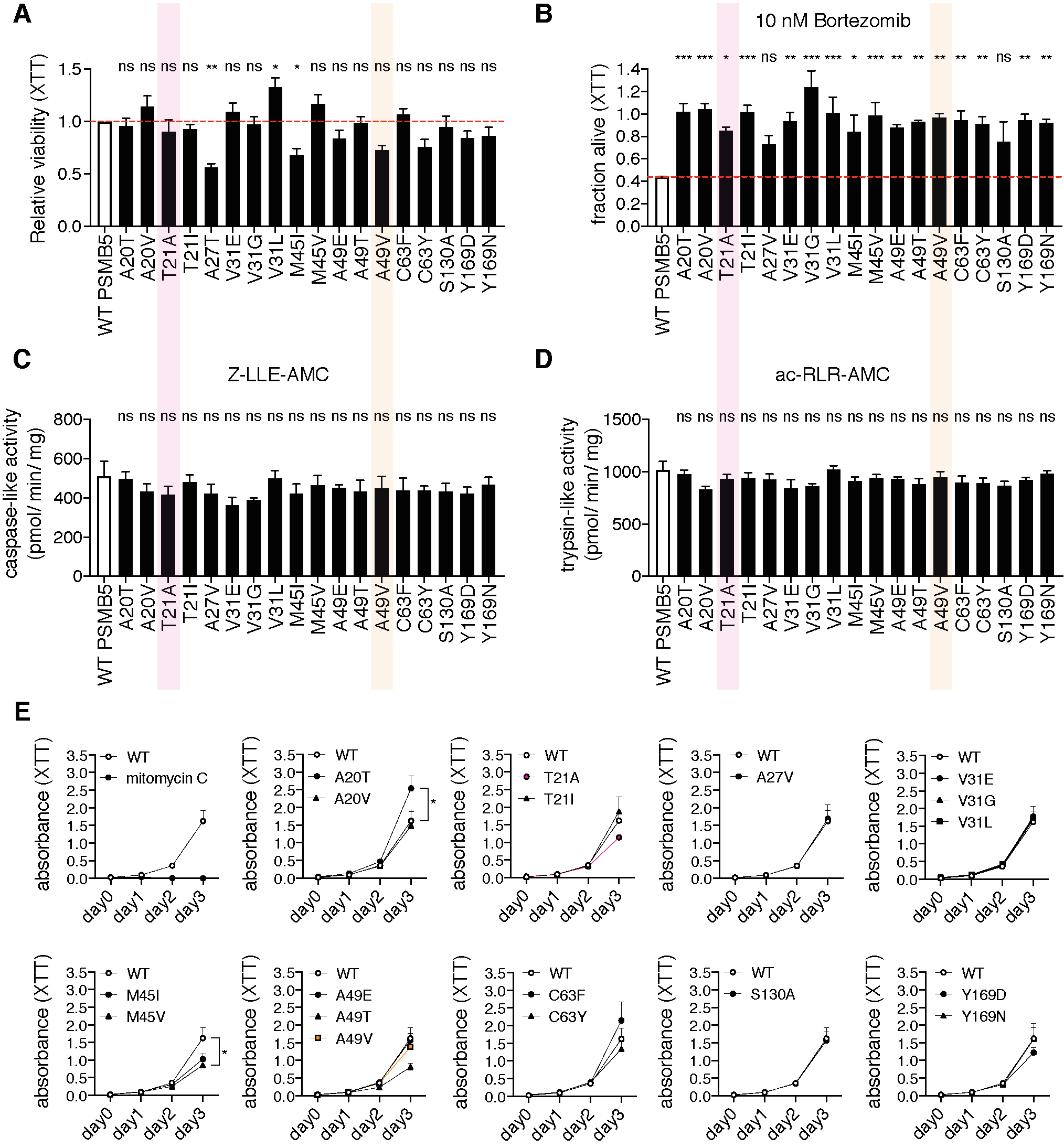


**Supplementary Figure 2: Functional characterization of Psmb5 mutant cells.** (**A**) Proliferation of Psmb5 mutant AN3-12 cell lines isolated from the bortezomib resistance screen and wildtype (WT) control cells assessed by cell viability assay. Mean + SEM (n=4). (**B**) Cell viability assay of CRISPR/Cas9-engineered AN3-12 cells with the indicated PSMB5 substitutions and wildtype control cells treated with 10 nM bortezomib. Mean + SEM (n=4). (**C**) Caspase-like proteasome activity of CRISPR/Cas9-engineered and wildtype control cells using Z-LLE-AMC as a substrate. Mean + SEM (n=3). (**D**) Trypsin-like proteasome activity of CRISPR/Cas9-engineered and wildtype control cells using ac-RLR-AMC as a substrate. Mean + SEM (n=3). (**A**-**D**) T21A is highlighted in pink, A49V is highlighted in orange. Statistical significance was calculated by One-way ANOVA Dunnett’s post-hoc test. *** p<0.001, ** p<0.01, * p<0.05, ns: not significant. (**E**) Proliferation of CRISPR/Cas9-engineered AN3‑12 cells with the indicated PSMB5 substitutions and wildtype control cells assessed by cell viability assay. Statistical significance was calculated by Two-way ANOVA, Fisher’s LSD test. * p<0.05. Data are presented as mean + SEM (n=4; n=3 for V31E/L and C63F/Y).


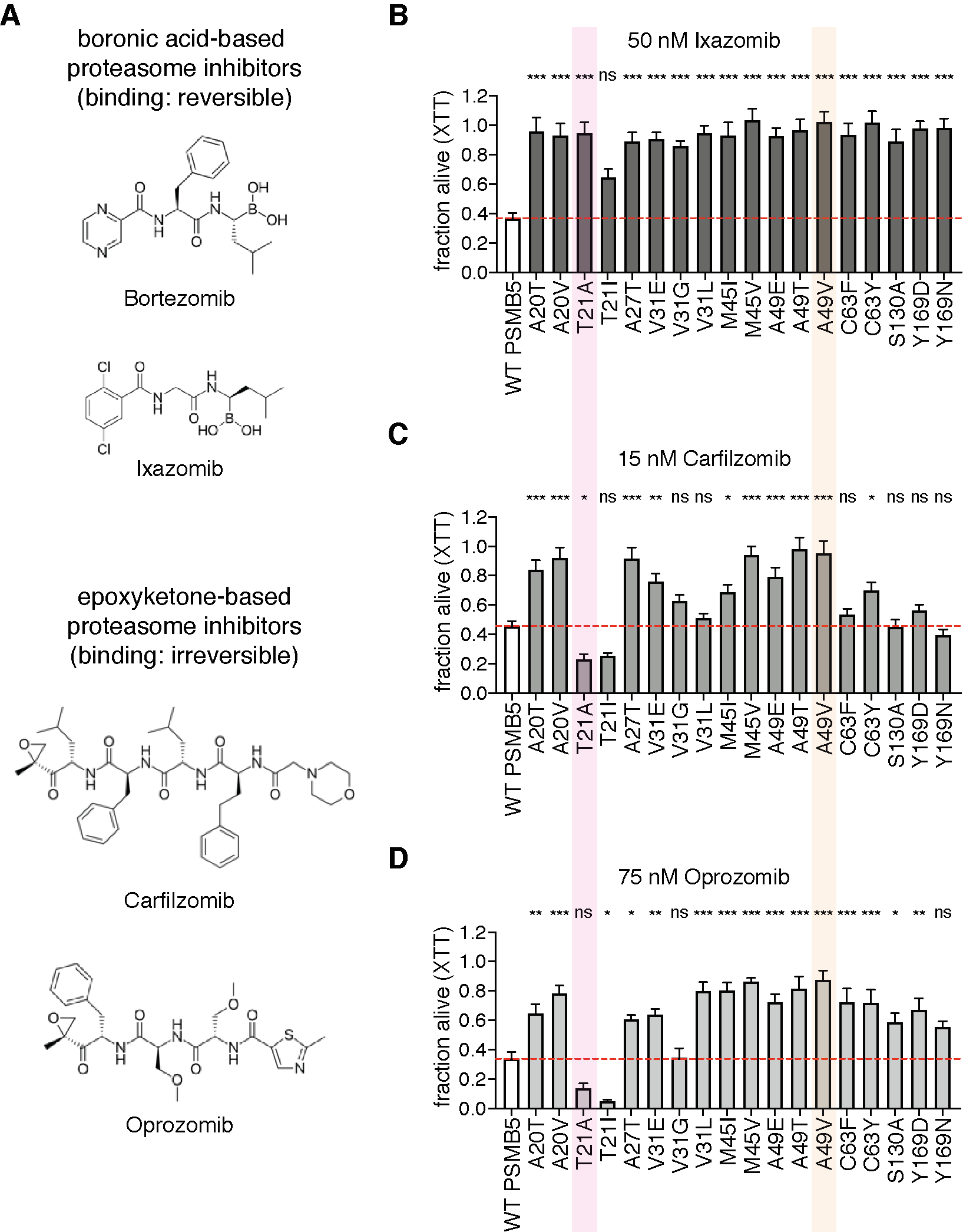


**Supplementary Figure 3: Psmb5 mutant cells isolated from the bortezomib resistance screen recapitulate resistance patterns of CRISPR/Cas9-engineered cells.** (**A**) Chemical structure of the PIs used in this study. (**B**) Cell viability assay of isolated AN3-12 clones with the indicated PSMB5 substitutions and wildtype control cells treated with 50 nM ixazomib. (**C**) Cell viability assay of isolated AN3-12 clones and wildtype control cells treated with 15 nM carfilzomib. (**D**) Cell viability assay of isolated AN3-12 clones and wildtype control cells treated with 75 nM oprozomib. (**B**-**D**) T21A is highlighted in pink, A49V is highlighted in orange. Statistical significance was calculated by One-way ANOVA Dunnett’s post-hoc test. *** p<0.001, ** p<0.01, * p<0.05, ns: not significant. Data are presented as Mean + SEM (n=4).


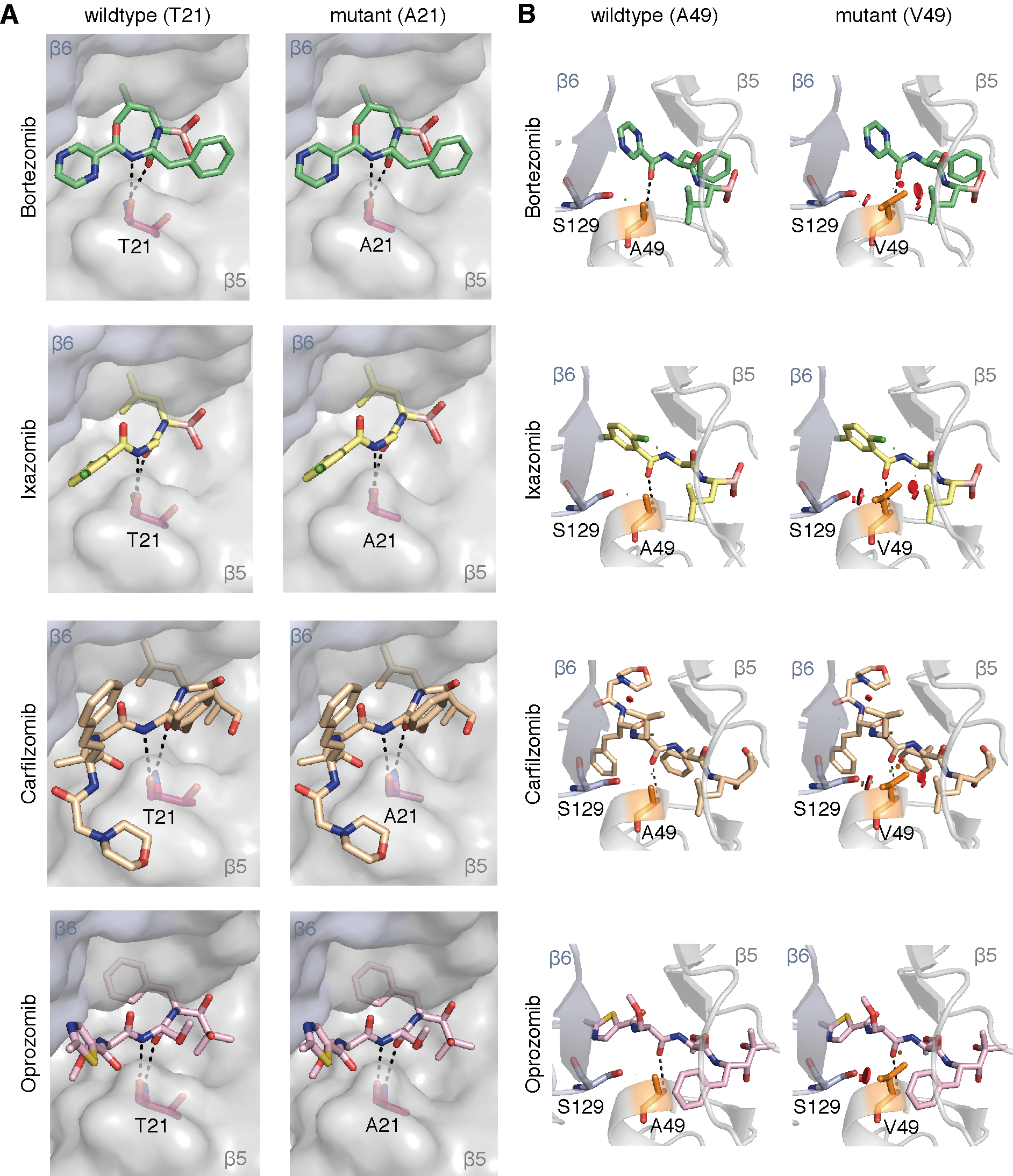


**Supplementary Figure 4: Resistance of PSMB5 T21A and A49V substitutions to different proteasome inhibitors can be explained on the structural level.** Crystal structure of human proteasome (β5 grey, β6 light blue) in complex with different PIs (bortezomib green, ixazomib yellow, carfilzomib light orange, oprozomib light pink). Hydrogen bonds are shown (black dashed lines) (**A**) T21 and A21 are highlighted in pink. (**B**) A49 and V49 are highlighted in orange. Steric clashes of V49 with S129 of the β6 subunit and PIs are shown as red discs (PyMOL plugin: show_bumps). PDB: 5LF3 (bortezomib), PDB: 5LF7 (ixazomib), PDB: 4R67 (carfilzomib), PDB: 5LEZ (oprozomib). The mutagenesis tool of PyMOL was used to model the substitutions.


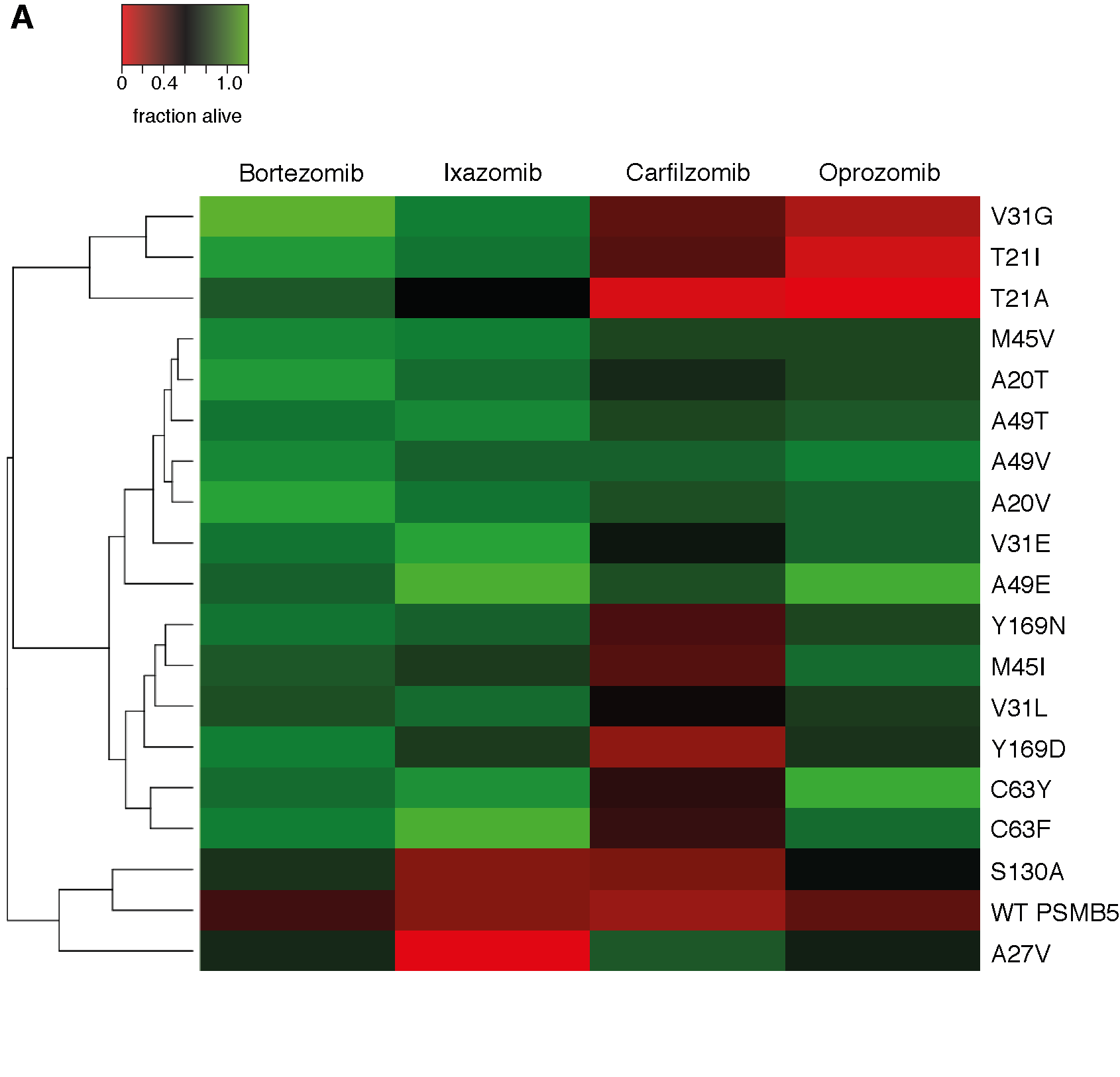


**Supplementary Figure 5: Heat map depicting observed resistance in CRISPR/Cas9-engineered AN3-12 cells with the indicated PSMB5 substitutions.** (**A**) Mean values of cell viability assays were entered into heatmapper (8) to obtain a cluster of the mutant cell lines according to their resistance against different PIs. Resistance is indicated by light green color. Black means comparable to WT. Red indicates sensitivity.

**Supplementary references**

1. Elling U, Taubenschmid J, Wirnsberger G, O'Malley R, Demers SP, Vanhaelen Q, et al. Forward and reverse genetics through derivation of haploid mouse embryonic stem cells. Cell Stem Cell. 2011;9(6):563-74.

2. Otsuki T, Yamada O, Sakaguchi H, Ichiki T, Kouguchi K, Wada H, et al. In vitro excess ammonia production in human myeloma cell lines. Leukemia. 1998;12(7):1149-58.

3. Otsuki T, Sakaguchi H, Hatayama T, Wu P, Takata A, Hyodoh F. Effects of all-trans retinoic acid (ATRA) on human myeloma cells. Leuk Lymphoma. 2003;44(10):1651-6.

4. Horn M, Kroef V, Allmeroth K, Schuller N, Miethe S, Peifer M, et al. Unbiased compound-protein interface mapping and prediction of chemoresistance loci through forward genetics in haploid stem cells. Oncotarget. 2018;9(11):9838-51.

5. Ran FA, Hsu PD, Wright J, Agarwala V, Scott DA, Zhang F. Genome engineering using the CRISPR-Cas9 system. Nat Protoc. 2013;8(11):2281-308.

6. Schrader J, Henneberg F, Mata RA, Tittmann K, Schneider TR, Stark H, et al. The inhibition mechanism of human 20S proteasomes enables next-generation inhibitor design. Science. 2016;353(6299):594-8.

7. Harshbarger W, Miller C, Diedrich C, Sacchettini J. Crystal structure of the human 20S proteasome in complex with carfilzomib. Structure. 2015;23(2):418-24.

8. Babicki S, Arndt D, Marcu A, Liang Y, Grant JR, Maciejewski A, et al. Heatmapper: web-enabled heat mapping for all. Nucleic Acids Res. 2016;44(W1):W147-53.
